# Supplementary material for: Causal Relationship between Aging and Anorexia Nervosa: A White-Matter-Microstructure-Mediated Mendelian Randomization Analysis
Source: Biomedicines. 2024 Aug 16;12(8):1874. doi: 10.3390/biomedicines12081874 (PMC11351342; doi:10.3390/biomedicines12081874)
Supplement: Supplementary file 1 [file biomedicines-12-01874-s001.zip › Supplementary Figure/Figure S4/Singe SNP plot of MR analysis for TL to ubm-b-2082.pdf]

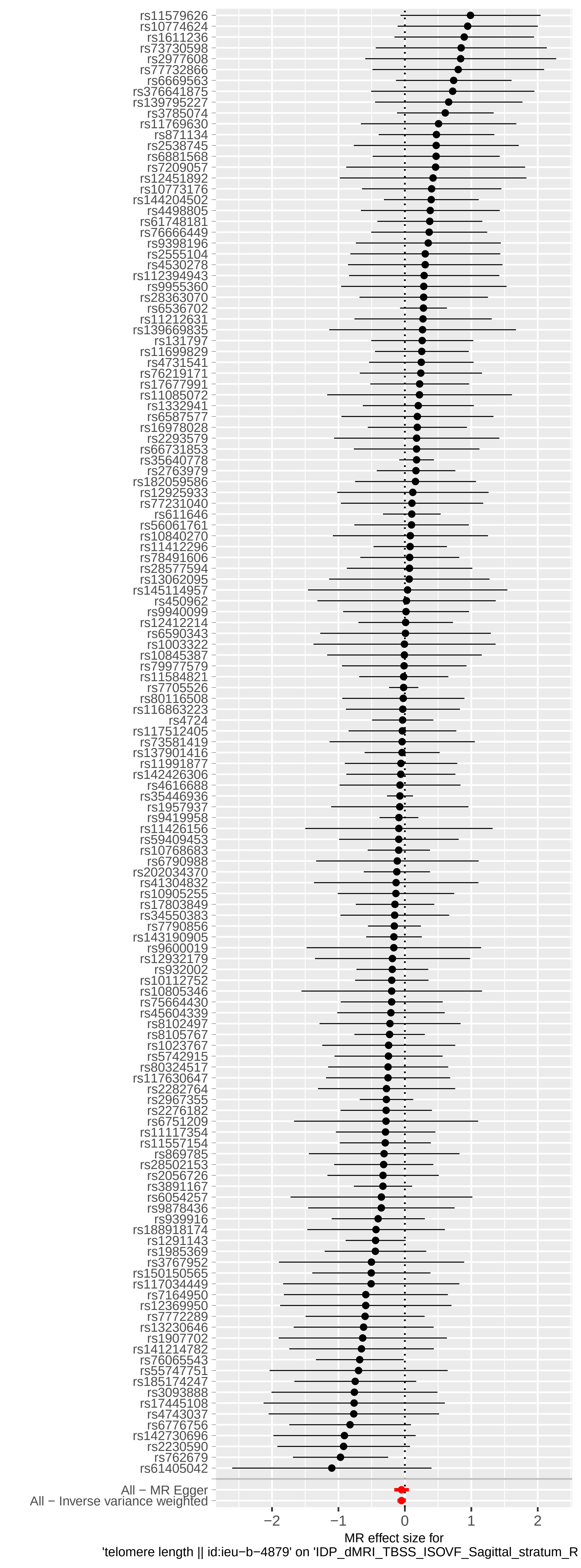

rs11579626
rs10774624
rs1611236
rs73730598
rs2977608
rs77732866
rs6669563
rs376641875
rs139795227
rs3785074
rs11769630
rs871134
rs2538745
rs6881568
rs7209057
rs12451892
rs10773176
rs144204502
rs4498805
rs61748181
rs76666449
rs9398196
rs2555104
rs4530278
rs112394943
rs9955360
rs28363070
rs6536702
rs11212631
rs139669835
rs131797
rs11699829
rs4731541
rs76219171
rs17677991
rs11085072
rs1332941
rs6587577
rs16978028
rs2293579
rs66731853
rs35640778
rs2763979
rs182059586
rs12925933
rs77231040
rs611646
rs56061761
rs10840270
rs11412296
rs78491606
rs28577594
rs13062095
rs145114957
rs450962
rs9940099
rs12412214
rs6590343
rs1003322
rs10845387
rs79977579
rs11584821
rs7705526
rs80116508
rs116863223
rs4724
rs117512405
rs73581419
rs137901416
rs11991877
rs142426306
rs4616688
rs35446936
rs1957937
rs9419958
rs11426156
rs59409453
rs10768683
rs6790988
rs202034370
rs41304832
rs10905255
rs17803849
rs34550383
rs7790856
rs143190905
rs9600019
rs12932179
rs932002
rs10112752
rs10805346
rs75664430
rs45604339
rs8102497
rs8105767
rs1023767
rs5742915
rs80324517
rs117630647
rs2282764
rs2967355
rs2276182
rs6751209
rs11117354
rs11557154
rs869785
rs28502153
rs2056726
rs3891167
rs6054257
rs9878436
rs939916
rs188918174
rs1291143
rs1985369
rs3767952
rs150150565
rs117034449
rs7164950
rs12369950
rs7772289
rs13230646
rs1907702
rs141214782
rs76065543
rs55747751
rs185174247
rs3093888
rs17445108
rs4743037
rs6776756
rs142730696
rs2230590
rs762679
rs61405042
All − MR Egger
All − Inverse variance weighted
−2
−1
0
1
2
MR effect size for
'telomere length || id:ieu−b−4879' on 'IDP_dMRI_TBSS_ISOVF_Sagittal_stratum_R
